# Administering Fluralaner in Drinking Water for Treatment of *Dermanyssus gallinae* Infestation in Hy-Line W80 Laying Hens

**DOI:** 10.5812/ijpr-145526

**Published:** 2024-06-02

**Authors:** MohammadReza Raeisi, Abdolkarim Zamani Moghaddam, Seyyed Sattar Tohidifar, Maryam Malekan

**Affiliations:** 1 Department of Clinical Sciences, Faculty of Veterinary Medicine, Shahrekord, Iran

**Keywords:** Poultry, Mite, Fluralaner, Miticide, Alternative Medicine

## Abstract

**Background:**

Poultry red mites, or *Dermanyssus gallinae*, pose a threat to the welfare and productivity of laying hens. Moreover, the increasing resistance of these mites to conventional miticides highlights the urgent need for alternative treatment options. There are also documented cases of poultry red mite infestations in humans.

**Objectives:**

The primary objective of this study was to evaluate the efficacy of fluralaner against *Dermanyssus gallinae* infestation in hens.

**Methods:**

Fluralaner was selected as a novel treatment for poultry red mite due to its effectiveness and safety profile. The presence of live mites in the drinking water tank served as the indicator of infestation. Live mites were counted on nine occasions throughout the study. Fluralaner was administered at three doses of 0.5 mg/kg in drinking water with a seven-day interval between each dose. The efficacy of fluralaner was assessed, with an efficacy percentage exceeding 90% considered indicative of antiparasitic efficacy.

**Results:**

The overall efficacy of Fluralaner in the current study exceeded 90% by day 5 and reached 100% by day 17.

**Conclusions:**

This study demonstrates that fluralaner is an effective alternative treatment, achieving efficacy rates exceeding 90% against poultry red mite infestation in laying hens.

## 1. Background

Poultry red mite (PRM), also known as *Dermanyssus gallinae*, poses a significant threat to poultry farms, particularly laying pullets, as it is an obligatory blood feeder. This exoparasite not only affects the welfare and productivity of both traditional and industrial poultry farms but also poses concerns for human health in residential areas, potentially irritating farm workers (1-12). Recent studies highlight a concerning increase in PRM incidence in Europe, with average rates reaching 83% among layers and peaking at 92% in countries like the Netherlands, Germany, and Belgium (6, 9). PRM incidence has been observed across all types of poultry flocks, including organic, layers, poultry meat, traditional, and backyard chickens (6). The rising concerns surrounding PRM have complicated its treatment, with conventional methods relying on miticides such as carbamates, organophosphates, amidines, pyrethroids, and more recently, spinosad in powder, mist, and spray forms (13-15). However, these conventional approaches face limitations such as difficulty in administering effective doses across all infested areas, causing additional stress to flocks, drug residue in birds and humans, and the emergence of miticide resistance (1, 13-18).

Fluralaner, an isoxazoline compound, is utilized as a systemic treatment for PRM. It functions as a potent inhibitor of the arthropod nervous system by antagonizing ligand-gated chloride channels (GABA-receptor and glutamate-receptor), which are abundant in the central and peripheral muscle-nervous systems of mites (19-21). This mechanism sets it apart from other miticides. Studies by Heckeroth et al. administered two doses of 0.5 mg/kg of Fluralaner with a one-week interval, resulting in an average reduction of the mite population by 99% fifteen days after the first administration (22). Additionally, Prohaczik et al. safely administered Fluralaner at a dose of 2.5 mg/kg repeatedly, further validating its efficacy (23). Following trials and the assessment of drug residue, Fluralaner received authorization for PRM treatment (24). 

## 2. Objectives

This study marks the first use of Fluralaner as a countermeasure against PRM in Iran, introducing a new index for grading PRM infestation.

## 3. Methods

Ninety laying pullets of Hy-Line W80, with an average age of 70 weeks, were housed in the poultry pen of the veterinary clinic at Shahrekord University. The poultry feed comprised corn and soybean, with birds receiving approximately 100 grams of feed per hen daily. To facilitate mite reproduction, excrements were collected less frequently, and live poultry red mites (*Dermanyssus gallinae*) were introduced to the flock. Despite being nocturnal, the mites were observed abundantly during the day, especially at the beginning of summer. A significant number of PRMs were observed clinging to a 15-liter water tank adjacent to the bird cages. The PRM infestation index was determined based on the number of live and mobile mites clinging to the outer surface of the water tank. Given that the study was conducted during the hottest month of the year, the mites were attracted to water and humidity.

**Figure 1.**
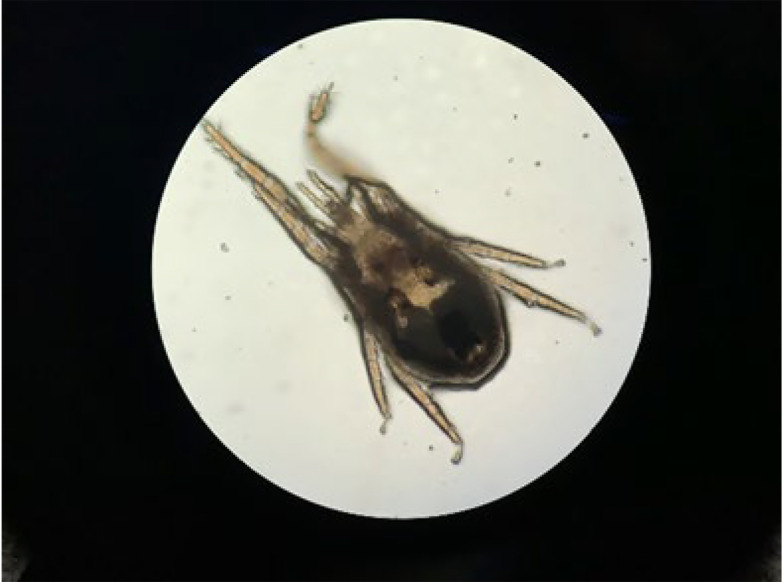
*Dermanyssus gallinae* isolated from the current study.

**Figure 2.**
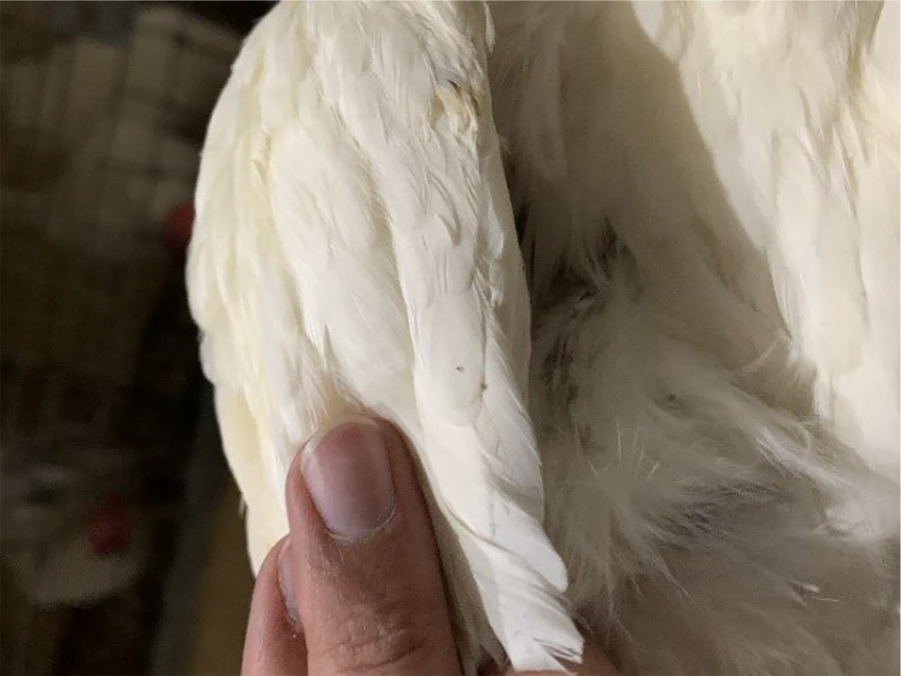
Red mites on the hens at night.

Three weeks before the main experiment, a preliminary experiment was conducted where a 2.5 mg/kg dose of Fluralaner was administered in the drinking water of 24 hens, and no adverse effects were observed, confirming previous studies suggesting the high margin of safety of Fluralaner (25). The main experiment aimed to determine the effectiveness of Fluralaner, with the chosen dose being 0.5 mg/kg. The average weight of the hens was 1200 grams, and 5.4 milliliters of a 1% Fluralaner solution were administered in the drinking water of the pen three times, with a 7-day interval. The water tank valve was closed to ensure the accurate administration of Fluralaner and opened only when the tank was empty. Figure 1 depicts a sample of *Dermanyssus gallinae* isolated from the infested hens, and Figure 2 shows a picture of a *Dermanyssus gallinae* mite on an infested hen at midnight.

### 3.1. Statistical Analysis

The antiparasitic efficacy of Fluralaner was considered achieved if the efficacy percentage exceeded 90%. The efficacy was calculated for each post-treatment day using the following formula.


$$\%efficacy=\frac{M\;\left(pre\right)-M\;(post)}{M\;(pre)}\times 100$$

## 4. Results

**Figure 3.**
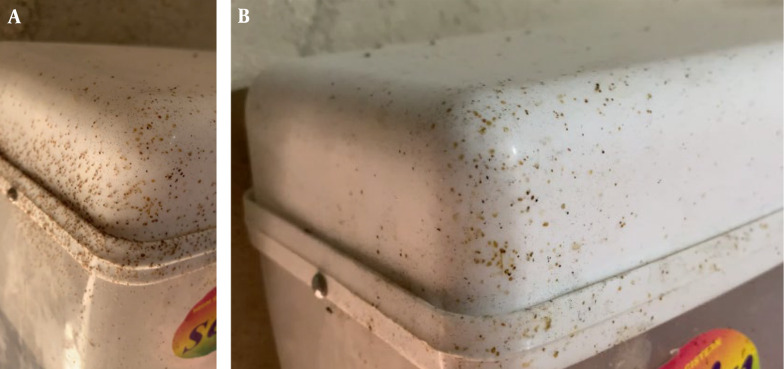
Before and after Fluralaner administration (top to bottom)

While few mites were detected on the birds, they were consistently observed on the water tank. Mites attached to the water tank were counted nine times during the 17-day experiment. Initially, mite infestation was significant, and they were widespread. Three doses of Fluralaner were added to the drinking water, and the counts of mites attached to the water tank are presented in Table 1. The overall efficacy of Fluralaner in the current study reached 100% by day 17 and exceeded 90% by day 5. Figure 3 depicts two pictures of the water tank before (a) and after (b) administration of Fluralaner.

**Table 1.** Fluralaner Administration and Mite Numbering

Mites	Action	Date
**152 mites were numbered**	Administration of first dose/numbering mites	7/2/2023
**67 mites were numbered**	Numbering mites	7/5/2023
**mites were numbered 43**	Numbering mites	7/6/2023
**mites were numbered 10**	Numbering mites	7/7/2023
**mites were numbered 23**	Administration of second dose/numbering mites	7/9/2023
**mites were numbered 19**	Numbering mites	7/11/2023
**11 mites were numbered**	Numbering mites	7/14/2023
**5 mites were numbered**	Administration of third dose/numbering mites	7/16/2023
**No mites were observed**	Numbering mites	7/18/2023

## 5. Discussion

Poultry red mite is a significant parasite globally, affecting egg production and animal welfare. Additionally, PRM poses a threat to human health, with reported cases of human infestation with PRM (26). In this study, Fluralaner was used as a new countermeasure against PRM. While the initial administration of Fluralaner significantly reduced mite numbers, efficacy decreased after the first week, necessitating a second dose. The elimination rate was lower in the second week, with mites persisting on the water tank. By the beginning of the third week, mite numbers were few but still present, prompting the administration of a third dose of Fluralaner. Two days after the third administration, no mites were detected attached to the water tank or inside the pen, indicating eradication. The primary challenge in eradicating PRMs is reaching antiparasitic medications to hard-to-reach locations such as grooves and hinges of cages. As PRMs are nocturnal and not present on the hens during the day, Fluralaner may affect them directly via water from the tank or through the blood from the hens. The slightly reduced efficacy of Fluralaner in this study may be attributed to the aging facility and cages. However, the antiparasitic efficacy of Fluralaner in the current study aligns with previous research.

**Figure 4.**
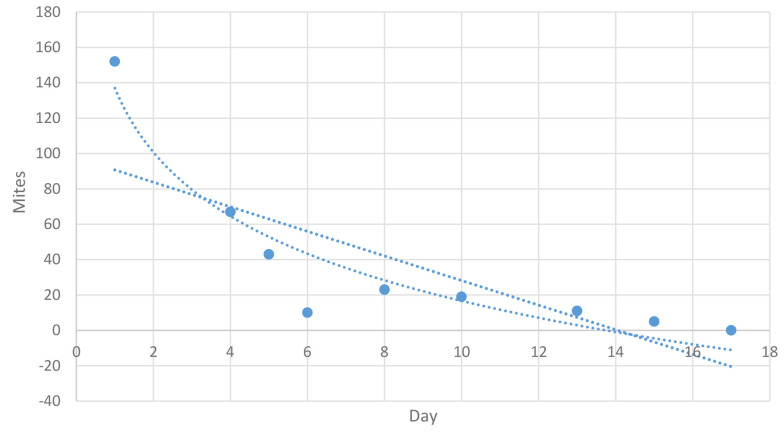
Mites attached to the water tank during 17 days of Fluralaner administration

Ivo Petersen et al., 2021, selected a PRM-infested free-range farm and a PRM-infested aviary farm. Fluralaner (Exzolt^®^; 0.5 mg/kg body weight) was administered twice, with a 7-day interval in drinking water. Throughout the post-treatment period, Fluralaner efficacy against PRM was > 99% on both farms (27). Notably, there was an unexpected increase in mites at day 7, probably due to the hatching of new larvae. The Full Life Cycle of *Dermanyssus gallinae* may last up to 11 days (6). The elimination rate and changes in mite numbers are analyzed in Figure 4. 

The R-squared value of 0.580 indicates that approximately 58% of the variability in the number of mites can be explained by the linear relationship with the day number. The P-value for the coefficient of the day variable is 0.020, which is less than the typical significance level of 0.05. This suggests that the day variable is statistically significant in predicting the number of mites.

### 5.1. Conclusions

Red poultry mites affect both chickens and humans. It is important to consider alternative medications to counter miticide resistance. Furthermore, Fluralaner can be administered via drinking water, making it easy to use and long-lasting with a high margin of safety. This underscores the utility of Fluralaner as an alternative treatment for PRM infestation. Three doses of 0.5 mg/kg of Fluralaner with a 7-day interval are required to eradicate PRM within a minimum period of 17 days.

## Data Availability

The dataset presented in the study is available on request from the corresponding author during submission or after publication.
